# Does Swimming Exercise Affect Experimental Chronic Kidney Disease in Rats Treated with Gum Acacia?

**DOI:** 10.1371/journal.pone.0102528

**Published:** 2014-07-21

**Authors:** Badreldin H. Ali, Suhail Al-Salam, Mohammed Al Za'abi, Khalid A. Al Balushi, Aishwarya Ramkumar, Mostafa I. Waly, Javid Yasin, Sirin A. Adham, Abderrahim Nemmar

**Affiliations:** 1 Department of Pharmacology and Clinical Pharmacy, College of Medicine and Health Sciences, Sultan Qaboos University, Alkhod, Muscat, Oman; 2 Department of Pathology, College of Medicine and Health Sciences, United Arab Emirates, Alin, United Arab Emirates; 3 Department of Food Sciences and Nutrition, College of Agricultural and Marine Sciences, Sultan Qaboos University, Alkhod, Muscat, Oman; 4 Department of Medicine, College of Medicine and Health Sciences, United Arab Emirates, Alin, United Arab Emirates; 5 Department of Biology, College of Science, Sultan Qaboos University, Sultan Qaboos University, Alkhod, Muscat, Oman; 6 Department of Physiology, College of Medicine and Health Sciences, United Arab Emirates, Alin, United Arab Emirates; University of Louisville, United States of America

## Abstract

Different modes of exercise are reported to be beneficial in subjects with chronic kidney disease (CKD). Similar benefits have also been ascribed to the dietary supplement gum acacia (GA). Using several physiological, biochemical, immunological, and histopathological measurements, we assessed the effect of swimming exercise (SE) on adenine –induced CKD, and tested whether SE would influence the salutary action of GA in rats with CKD. Eight groups of rats were used, the first four of which were fed normal chow for 5 weeks, feed mixed with adenine (0.25% w/w) to induce CKD, GA in the drinking water (15% w/v), or were given adenine plus GA, as above. Another four groups were similarly treated, but were subjected to SE during the experimental period, while the first four groups remained sedentary. The pre-SE program lasted for four days (before the start of the experimental treatments), during which the rats were made to swim for 5 to 10 min, and then gradually extended to 20 min per day. Thereafter, the rats in the 5^th^, 6^th^, 7^th^, and 8^th^ groups started to receive their respective treatments, and were subjected to SE three days a week for 45 min each. Adenine induced the typical signs of CKD as confirmed by histopathology, and the other measurements, and GA significantly ameliorated all these signs. SE did not affect the salutary action of GA on renal histology, but it partially improved some of the above biochemical and physiological analytes, suggesting that addition of this mode of exercise to GA supplementation may improve further the benefits of GA supplementation.

## Introduction

Chronic kidney disease (CKD) is a major public health concern in both developed and developing countries because of the high prevalence of morbidity and mortality associated with it, mainly due to cardiovascular dysfunction [1], [2]. It has been suggested that CKD leads to reduced physical activity and an increased risk of cardiovascular disease (CVD) [2], [3]. A sedentary lifestyle increases the risk of CVD, but CVD can be ameliorated by physical fitness [3], [4], [5].

Aerobic exercise has been shown to improve renal and cardiac function in individuals with CKD [6] and in overweight rats with metabolic and cardiac dysfunction [7], and exercise has gained more attention as a possible tool for preventing, reducing or delaying CKD progression [3], [4], [5], [6], [7]. It has been suggested that appropriate exercise may improve a patient's physical strength and quality of life [8], [9]. Swimming has been increasingly prescribed as a non-pharmacological treatment for arterial hypertension, obesity and coronary heart disease [10], [11]. Thus, improving our knowledge of the effects of swimming training in animal models is relevant for CKD patients [5], [6], [12], [13], [14].

Because of the rise in recent decades of CKD incidence and its associated cardiovascular risks and damage [15], we thought it of importance to assess the effect of swimming exercise on a relevant rodent model of human CKD [16], and further, to evaluate the effect of co- administration of a natural product, gum acacia, which has recently been shown to ameliorate CKD in patients [17], [18] and rats [19], [20], [21], [22], [23].

## Methods

### Animals

Male Wistar rats (9–10 weeks old, weighing 249±10 g) were housed in a room at a temperature of 22±2°C, relative humidity of about 60%, with a 12 h light–dark cycle (lights on 6 00), and free access to standard pellet chow diet containing 0.85% phosphorus, 1.12% calcium, 0.35% magnesium, 25.3% crude protein and 2.5 IU/g vitamin D3 (Oman Flour Mills, Muscat, Oman) and water. Ethical approval of this work was obtained from our University Animal Research Ethics committee, and all procedures involving animals and their care were carried out in accordance with international laws and policies (EEC Council directives 86/609, OJL 358, 1 December, 12, 1987; NIH Guide for the Care and Use of Laboratory Animals, NIH Publications No. 85–23, 1985), and ethical clearance was obtained from the Small Animal Research Ethics Committee of Sultan Qaboos University.

### Experimental Design

After an acclimatization period of one week, rats (n = 48) were randomly divided into eight equal groups and treated for five consecutive weeks. The 1st group continued to receive the same diet without treatment until the end of the study (control group). The 2^nd^ group was switched to a powder diet containing adenine (0.25%w/w in feed for 5 weeks). The 3^rd^ group was given normal food and GA in drinking water at a concentration of 15% w/v for 5 weeks. The 4^th^ group was given adenine in the feed as in group two, plus GA in drinking water at a concentration of 15% w/v. The dose of adenine was chosen from previous reports [19]–[21]. The 5^th^, 6^th^, 7^th^ and 8^th^ groups were treated in the same manner as the 1^st^, 2^nd^, 3^rd^ and 4^th^ group, respectively, except that these latter four groups were also subjected to swimming exercise (SE) (see below)

### Swimming Exercise (SE) Training Protocol

Rats were subjected first to a pre-SE for acclimation in an experimental swimming pool (∼30°C, water depth: 30 cm; radius 120 cm), as described by others [14], [22], [24]. The pre SE program lasted for an acclimation period of four days (before the start of the experimental treatments), during which the rats were made to swim for 5 to 10 min, and then gradually extended to 20 min per day. After the acclimation to swimming, the rats in the 5^th^, 6^th^, 7^th^ and 8^th^ groups started to receive their respective treatments, and were subjected to SE three days a week for 45 min each.

### Treatments

During the treatment period, the rats were weighed weekly. For the collection of urine, they were placed individually in metabolic cages for 24 h, after the 35 days treatment period. On the morning after the metabolic sampling, the rats were anesthetized with an intraperitoneal injection of ketamine (75 mg/kg) and xylazine (5 mg/kg), and blood (about 4 mL) was collected from the anterior vena cava and placed into heparinized tubes. The blood and urine were centrifuged at 900 *g* at 4°C for 15 min. The plasma obtained, together with the urine specimens, was stored at −80°C to await analysis within 4 weeks after the end of the treatment. The two kidneys were excised, blotted on filter paper and weighed. A part of the right kidney was placed in formalin, awaiting histopathological studies. The rest of the kidneys were kept frozen at −80°C pending biochemical analysis within three days. The left kidney was homogenized in ice-cold Tris buffer (pH 7.4) to give a 10% w/v homogenate. The latter was centrifuged at 1500 *g* at 4°C for 15 min, and the supernatant obtained was used to measure superoxide dismutase (SOD) and catalase (CAT) activities, the concentrations of glutathione (GSH), and total antioxidant capacity (TAC).

### Biochemical and Physiological Measurements

Traditional and novel biochemical urinary, plasma and renal biomarkers were measured. Creatinine, urea, uric acid, calcium (Ca), phosphorus (P) and protein concentrations in plasma and/or urine were measured spectrophotometrically using commercial kits. In renal cortex homogenates, protein concentration was measured by Lowry's method using albumin as a standard. TAC, and GSH concentration, as well as CAT and SOD activities in plasma, and urinary 8-oxo-2'-deoxyguanosine(8-OHDG) were measured using ELISA kits, as described before [19]–[22].

In plasma, nephrin, tumor necrosis factor α (TNFα), 8-isoprostane, adiponectin and cystatin C were measured using ELISA - based commercial kits. The uremic toxin indoxyl sulfate was measured by a validated HPLC method developed in this laboratory [25].

### Histopathology

After weighing, the kidneys were sampled and fixed in 10% neutral-buffered formalin for 24–48 hrs, dehydrated in increasing concentrations of ethanol, cleared with xylene and embedded in paraffin. Four micrometer (µm) sections were prepared from kidney paraffin blocks and stained with hematoxylin and eosin (H & E). The microscopic scoring of the kidney sections was carried out in a blinded fashion by a pathologist who was unaware of the treatment groups, and assigned a score, as described before [19], which represents the approximate extent of the necrotic area in the cortical and medullary tubules, and assigned a score on a scale of 0–4 (0, no necrosis; 1, a few focal necrotic areas of ≤25% of the kidney; 2, necrotic area was about 26–50% of kidney; 3, necrotic area was 51–75% of kidney; 4, nearly the entire area was necrotic, necrotic area was 76–100% of kidney).

The size of the necrosis was also estimated, and values were presented as means ± SEM.

Four-µm sections were prepared from paraffin blocks and stained with Masson trichrome stain to assess the degree of interstitial fibrosis. Image J software (NIH, USA) was used to measure the extent of necrosis and fibrosis.

Staining for apoptosis was performed with a signal stain-cleaved caspase-3immuno-histochemical detection kit. This was used to detect the activation of caspase using the avidin–biotin immunoperoxidase method to detect intracellular caspase-3 protein. Staining was performed on 5 µm paraffin sections from the left kidney by a standard technique using rabbit anti-cleaved caspase 3 (clone Asp175, 1∶50) [16]. Known positive control sections for apoptosis were used. For negative control, primary antibody was replaced with normal rabbit serum. The apoptotic index was calculated by dividing the number of positive tubular epithelial cells for anti-casapase-3 per 100 tubular epithelial cells. The calculation was repeated in at least 10 random high power fields and the total was divided by 10 to get the apoptotic index.

### Western blot analysis for caspase-3 and its cleaved isoform

Since caspase cascade activation is a known feature of apoptosis which is associated with CKD [16], we measured here the proteolytic activity of caspase-3 in the rat kidneys collected from the eight different groups. The kidneys were homogenized by crushing 0.5 µg of the tissue using a micro size mortar and pistol in cold lysis buffer (Cell Signaling Technologies, USA) containing protease inhibitor cocktail (Sigma, Aldrich, USA). Kidney lysates were centrifuged and quantified using BCA protein assay system (Pierece, USA). Aliquots of total protein from each sample (100 µg) were loaded into a 15% SDS-PAGE gel. Protein was transferred to PVDF membrane (Millipore, Belgium). The membranes were blocked with 5% nonfat milk in TBST (10 mM Tris, pH 7.5, 150 mM NaCl, 0.05% Tween 20) and probed with 1∶1000 dilution of caspase-3 primary monoclonal rabbit antibody which was prepared to detect both caspase-3 bands, not cleaved (37 KDa) and cleaved band (25 KDa) (Cell Signaling Technology, USA) in the same blot. The antibody was added to 5% nonfat milk/TBST solution. Immunoblots were then processed with horseradish–peroxidase-conjugated anti-rabbit immunoglobulin G (IgG) (secondary antibody using the enhanced BM Chemiluminescence Western Blotting Kit (Mouse/Rabbit) (Roche, USA). The membranes were stripped off and re- blotted using beta Actin primary antibody (Cat # 4970 from Cell Signaling Technology, USA). The blots were exposed to X-ray film (Roche, U.S.A) at room temperature. Densitometery was carried out on the scanned X-ray film using Image J software which measures the relative intensity of the test band in respect to the loading control beta actin.

### Drugs, Chemicals and Kits

GA used was SUPERGUMTMEM10, Lot 101008, 1.1.11 (San – Ei Gen F. F. I.; Sanwa-Cho, Toyonaka, Osaka, Japan); aqueous solutions were prepared freshly every day. The chemical properties of GA have been fully reported before [20], [21]. The SUPERGUMTM EM 10 used was characterized by size fractionation followed by multiple angle laser light scattering (GPC-MALLS) to give its molecular profile. The average molecular weight was 3.43×106, and the content of arabinogalactan protein (AGP) 26.4%. Adenine was obtained from Sigma (St. Louis, MO, USA). Creatinine, urea and protein kits were bought from Human GmbH (Mannheim, Germany) and SOD, CAT and AO kits from Randox (Antrim, UK). TAC kits were from Cayman Chemical, Ann Arbor, MI, USA. Nephrin was obtained from Novatein Biosciences, Cambridge, MA, USA, tumor necrosis factor α (TNF α) from Cayman Chemical, Ann Arbor, MI, USA, 8-isoprostane and 8-oxo-2'-deoxyguanosine (8-OHDG) from Statok Kino, Shizuoka, Japan, adiponectin from Cayman Chemical, Ann Arbor, MI, USA, and cystatin C from R &D Systems, Abingdon, UK.

### Statistical Analysis

All data were analyzed with GraphPad Prism Version 4.01 for Windows software (Graphpad Software Inc., San Diego, CA). Data were analyzed for normal distribution using the D'Agostino and Pearson omnibus normality test. Data are expressed as means ± SEM.

Comparisons between groups were performed by one-way ANOVA, followed by Newman- Keuls test for comparing treated with control data; *P* values of less than 0.05 are considered significant.

## Results

### Physiological Results

**Figure 1:**
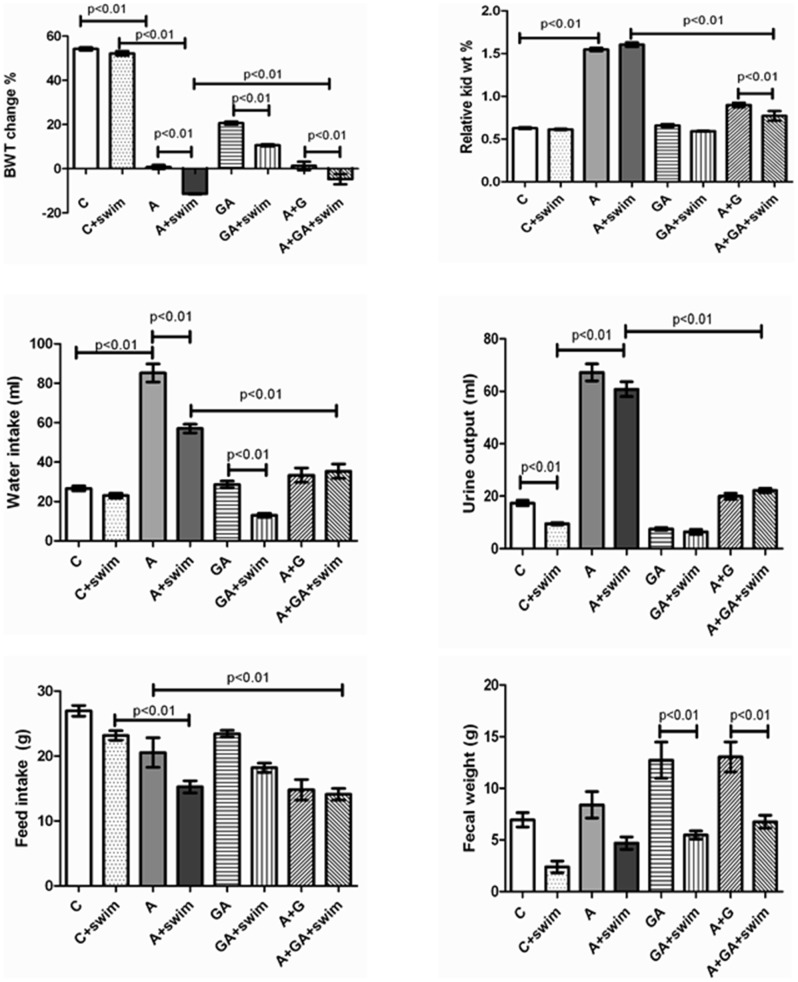
Body weight change (%), relative kidney weight (%), water intake, urine volume, feed intake, and fecal weight in rats treated with saline (C); saline + swimming exercise, (C + swim); adenine (A); A + swim; gum acacia (GA); GA + swim; A + GA; and A + G + swim. Each column is mean ± SEM (n  =  six rats). Statistical analysis by ANOVA followed by Newman– Keuls test.

Rats that had undergone SE in the different groups appeared more active than their sedentary counterparts. As shown in Fig 1, SE did not significantly change body weight of control rats, but it significantly reduced that of rats with CKD. Treatment with GA reduced the body weight, an effect that was potentiated by SE. When GA treatment was combined with SE and adenine, the body weight of rats was depressed even further.

The weights of the kidneys relative to the final body weight of adenine –treated rats were significantly higher than those of the control rats. This action was not significantly affected by the SE.

Water intake and urine volume in the adenine –treated rats were significantly higher than in control rats (*P*<0.05), and this was significantly abated by SE and GA treatment.

Feed intake but not fecal weight was reduced by adenine treatment. In all groups SE reduced both the feed intake and fecal weight.

### Biochemical Results

**Figure 2:**
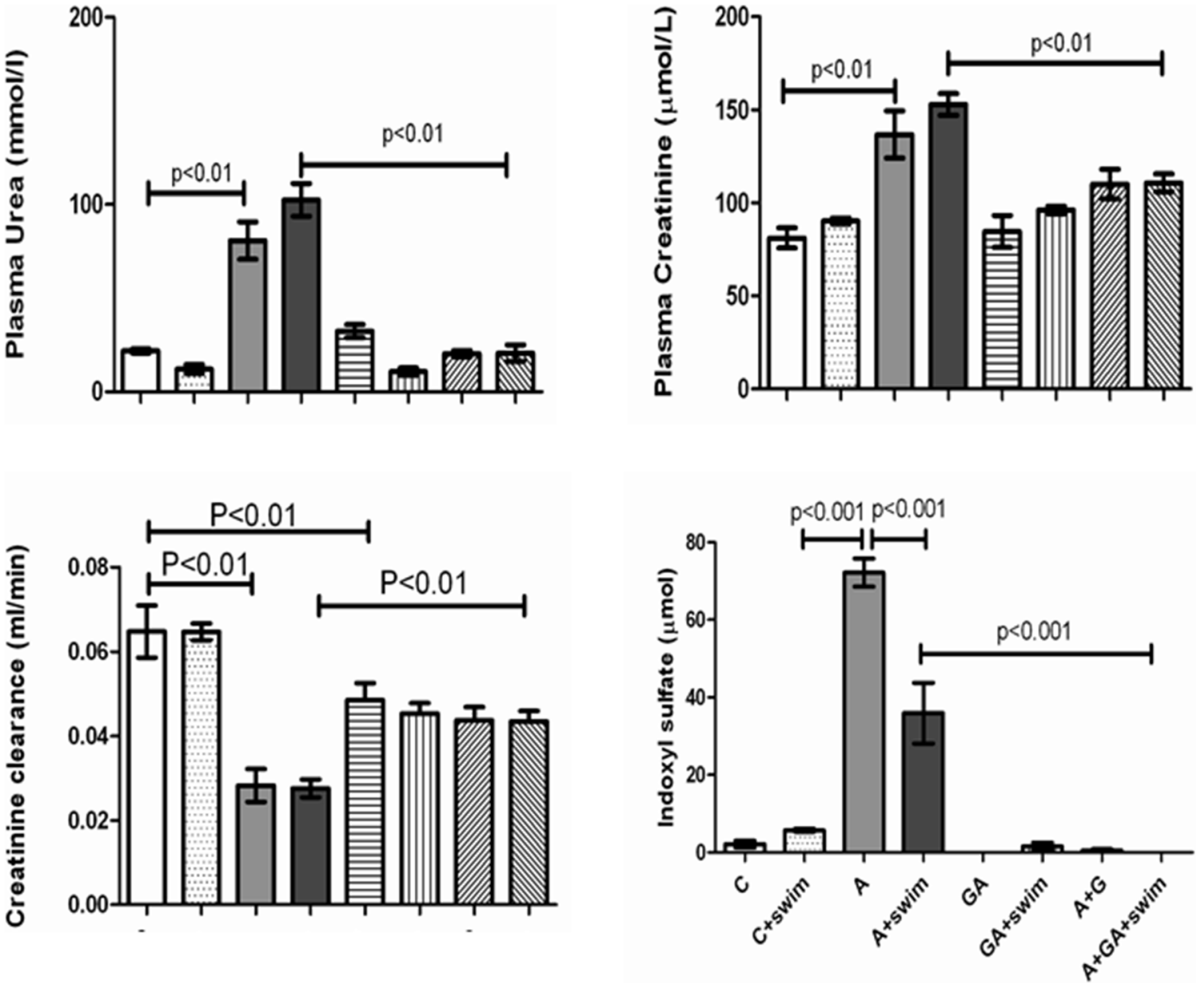
The plasma concentrations of urea, creatinine and indoxyl sulfate, and the creatinine clearance in rats treated with saline (C); saline + swimming exercise, (C + swim); adenine (A); A + swim; gum acacia (GA); GA + swim; A + GA; and A + G + swim. Each column is mean ± SEM (n  =  six rats). Statistical analysis by ANOVA followed by Newman–Keuls test.

Fig 2 shows the plasma concentrations of indoxyl sulfate, creatinine and urea, as well as the creatinine clearance in the eight groups. Adenine treatment significantly increased the concentrations of indoxyl sulfate, creatinine and urea, and decreased that of the creatinine clearance. This effect was significantly but not completely reversed by GA treatment. Concomitant SE did not significantly affect any of the above analytes.

**Figure 3:**
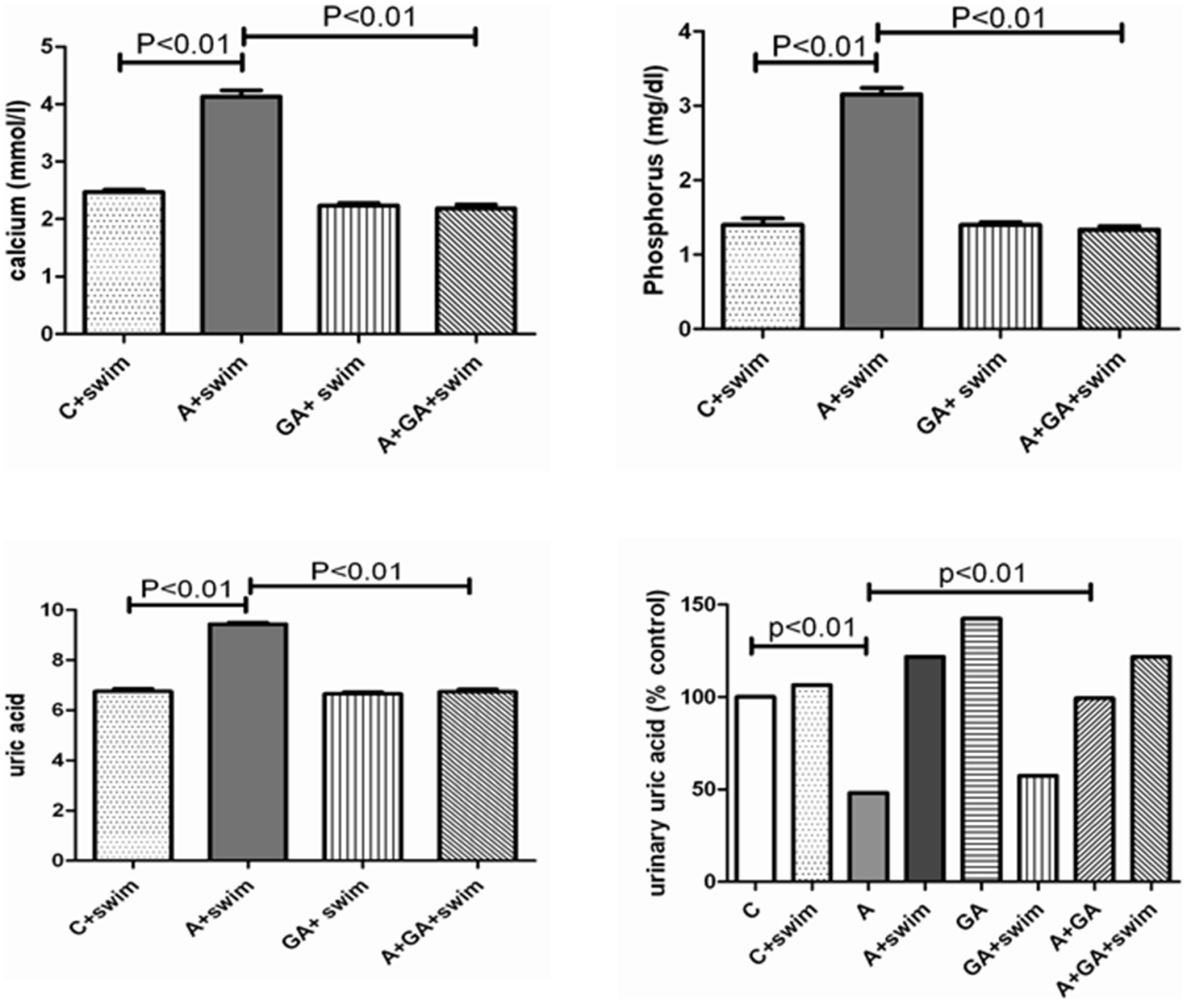
The effect of treatment with saline (C); saline + swimming exercise (C + swim); adenine (A); A + swim; gum acacia (GA); GA + swim; A + GA; and A + G + swim on urinary uric acid concentration, and the plasma concentration of calcium, phosphorus and uric acid in treated with saline, A, GA and A + GA in rats subjected to swimming exercise. Each column is mean ± SEM (n  =  six rats). Statistical analysis by ANOVA followed by Newman–Keuls test.

Adenine treatment significantly decreased Ca, but increased P and uric acid concentrations (data not shown). However, as shown in Fig 3, in rats similarly treated but subjected to SE, Ca and uric acid concentrations were significantly increased, and P remained higher than the control (sedentary and subjected to SE). SE in control rats had no significant effect on urinary uric acid excretion, but in adenine –treated rats SE induced a significant rise (*P*<0.01). The adenine – induced significant decrease in urinary uric acid excretion was significantly (*P*<0.01) but not completely antagonized by GA treatment.

**Figure 4:**
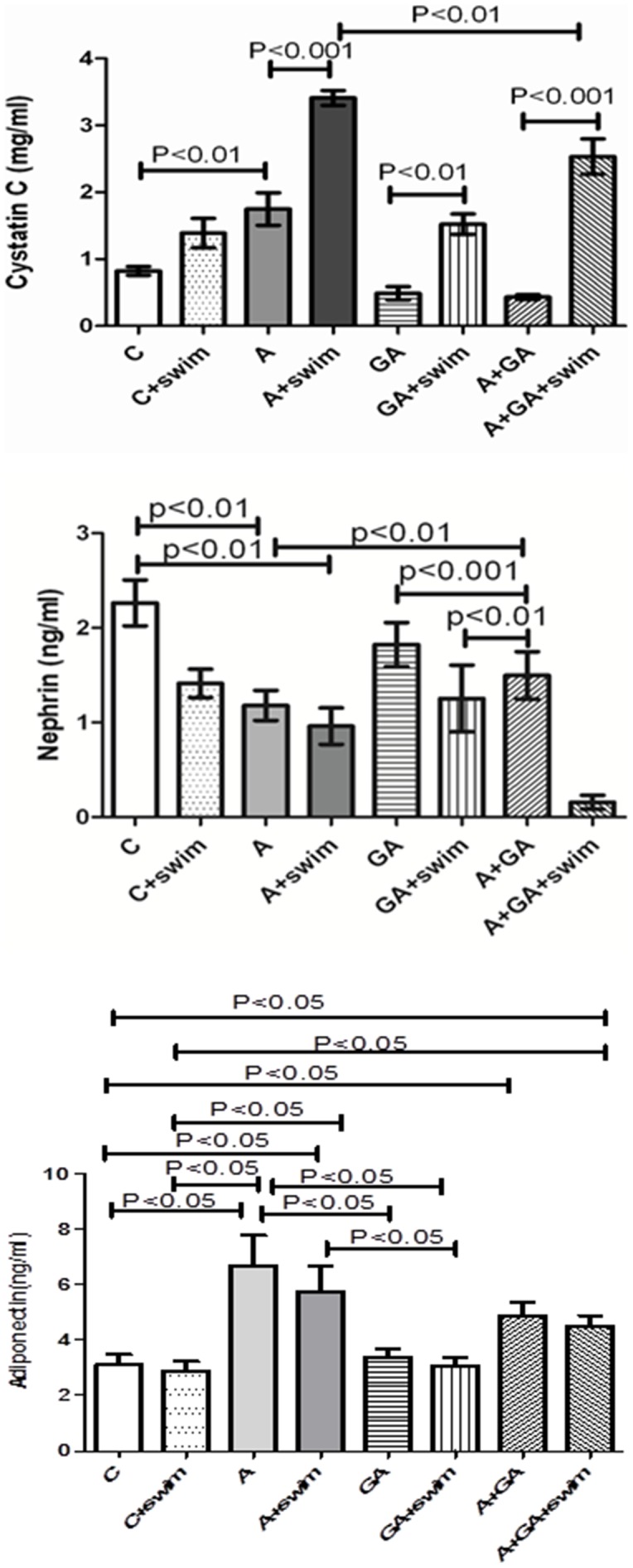
The concentrations of cystatin C, nephrin and adiponectinin plasma of rats treated with saline (C); saline + swimming exercise (C + swim); adenine (A); A + swim; gum acacia (GA); GA + swim; A + GA; and A + G + swim. Each column is mean ± SEM (n  =  six rats). Statistical analysis by ANOVA followed by Newman–Keuls test.

The effects of SE and GA treatments in adenine –treated rats on Cystatin C, nephrin and adiponectin concentrations in plasma are shown in Fig 4. Adenine treatment significantly increased the concentration of cystatin C, while GA caused the opposite effect. However, in all the treated groups, SE significantly increased the concentration of cystatin C. The plasma nephrin concentration was significantly reduced by adenine treatment, an effect which was further enhanced by SE in all groups. Adenine treatment significantly increased adiponectin concentration, and this was not significantly affected by SE in any of the groups (Fig 4).

The effect of SE and GA treatments in adenine –treated rats on the concentration of some proteins, cytokines and antioxidants in plasma is shown in Table 1.

**Table 1.The:** effect of swimming exercise (SE) on some cytokines, antioxidant markers and proteins in plasma or urine from rats with chronic kidney disease induced by feeding adenine (A) [0.25% w/w, 5 weeks], and the influence of gum acacia (GA) [15% w/v in drinking water, 5 weeks] thereon.

Groups	Plasma TNFα (pg/mL)	Plasma 8-isoprostone (pg/mL)	Urine OHdG (ng/mL)	Plasma adiponectin (ng/L)	Plasma cystatin C (pg/L)	Plasma nephrin (pg/mL)
Control (C)	49.3±3.2^a^	51.0±3.9^a^	181.2±14.7^a^	3.7±0.2^a^	0.55±0.03^a^	0.47±0.02^a^
C + SE	47.7±4.0^a^	47.2±3.8^a^	178.9±15.9^a^	3.3±0.2^a^	0.57±0.03^a^	0.45±0.03^a^
A	66.9±6.1^a^	83.4±7.5^a^	223.1±19.2^a^	6.1±0.4^b^	0.87±0.06^a^	0.70±0.05^b^
A + SE	54.0±4.2^a^	61.2±4.3^a^	190.1±15.3^a^	5.7±0.3^b^	0.64±0.05^a^	0.65±0.02^c^
GA	48.3±4.0^a^	50.7±5.2^a^	172.3±16.3^a^	3.9±0.3^a^	0.48±0.04^a^	0.46±0.02^a^
GA + SE	45.9±4.2^a^	47.9±5.0^a^	169.9±17.1^a^	3.6±0.3^a^	0.5±0.05^a^	0.44±0.03^a^
A + GA	56.1±4.8^a^	59.2±4.7^a^	188.1±15.3^a^	4.7±0.4^c^	0.66±0.06^a^	0.6±0.05^c^
A + GA + SE	52.1±4.9^a^	55.2±4.1^a^	179.2±15.4^a^	4.0±0.3^a^	0.59±0.05^a^	0.55±0.04^d^

Values in the table are means ± SEM (n = 6 rats). Values with similar superscripts are not statistically different (level of significance set at *P*<0.05).

TNFα  =  Tumor necrosis factor alpha; 8- OHdg  = 8 – hydroxo – 2- deoxyguanisone;

The effect of SE and GA treatment in adenine –treated rats on indices of oxidative damage is shown in Table 2. Compared with control sedentary rats, SE raised the four indices of oxidative damage measured, which was only statistically significant in the case of SOD activity (where it was raised by 17%, *P*<0.5). Adenine treatment significantly decreased the four indices, and SE in adenine –treated rats insignificantly and incompletely reversed that action. Treatment of rats with either GA alone or together with SE had no significant effect on any of the indices of oxidative damage. GA treatment significantly restored these indices to near normal levels, and this action was not significantly affected by SE.

**Table 2:** The effect of swimming exercise (SE) on some antioxidant indices in kidney homogenates from rats with chronic kidney disease induced by feeding adenine (A) [0.25% w/w, 5 weeks], and the influence of gum acacia (GA) [15% w/v in drinking water, 5 weeks] with or without SE thereon.

Groups	SOD µmol/min/mg protein	CAT µmol/min/mg protein	GSH nmol/mg protein	TAC nmol/mg protein
Control (C)	66.6±4.1	90.7±6.3	27.6±1.8	118.2±6.4
C + SE	77.7±4.9*	115.2±5.1	31.6±2.0	136.3±7.5
A	35.2±0.6**	28.4±4.8**	11.7±3.4**	68.3±3.9**
A + SE	37.8±1.4**	35.8±1.5**	13.6±1.3**	76.9±2.8**
GA	69.7±3.1	108.3±7.1	29.8±2.2	123.6±5.9
GA + SE	73.3±4.0	110.4±6.0	30.7±1.9	133.9±5.3*
A + GA	65.3±4.3	88.9±3.1	27.1±1.9	117.9±5.1
A + GA + SE	69.2±3.0	96.3±4.9	28.4±1.5	124.0±4.5

Values in the table are means ± SEM (n = 6 rats).

SOD  =  Superoxide dismutase; CAT  =  catalase; GSH  =  reduced glutathione and TAC  =  total antioxidant capacity.

*P less than 0.05.

**P less than 0.01 (Compared to control for the same index).

### Western Blotting Results

**Figure 5:**
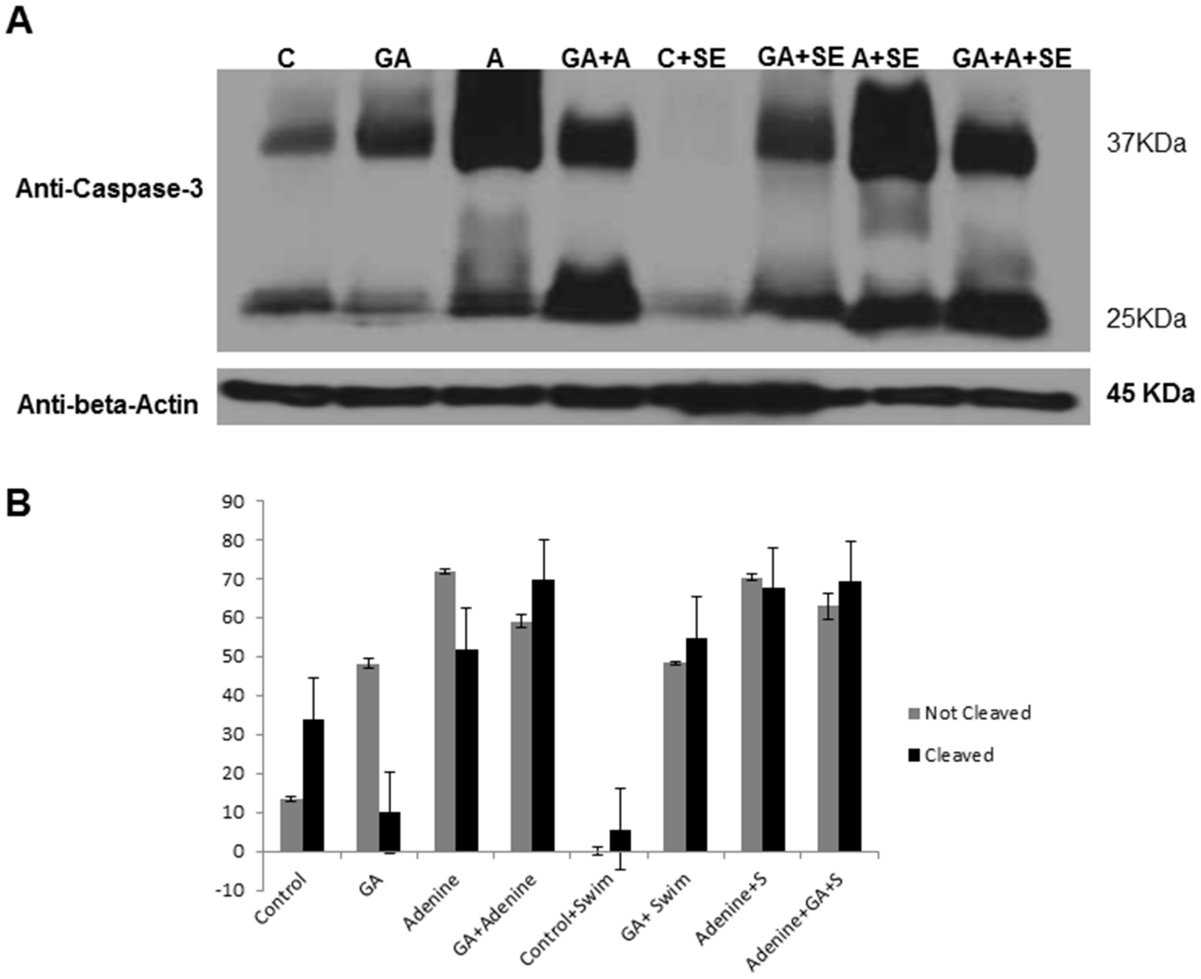
Representative photograph and quantification of apoptosis and caspase 3 cleavage in kidney tissue lysates of rats treated with saline (1); saline + swimming exercise, SE (2); adenine (3); adenine + SE (4);gum acacia, GA (5); GA + SE (6); adenine (3), adenine + GA (7); and adenine + GA + SE (8). Columns and vertical bars represent means ± SEM of relative intensity of cleaved and uncleaved bands. Western blotting is depicted for caspase-3, cleaved caspase-3 and β-Actin loading protein.

Western blot densitometric quantitation (Fig 5) showed that SE significantly decreased the activity of caspase-3 cleavage only in the control and not in the treated rats (*P = *0.004). However, SE significantly increased caspase-3 activity in animals treated with either adenine alone, or GA alone (*P = *0.0016, *P = *0.0004). SE did not result in any significant difference in caspase-3 cleavage in the group that had been treated with both adenine and GA.

### Histopathological and Immunohistochemical Results

**Figure 6:**
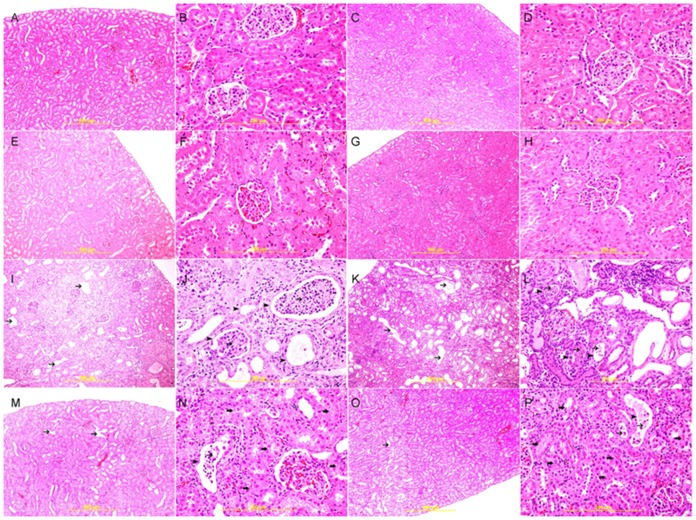
Representative photograph of sections of renal tissue of rats treated with saline (A), saline + swimming exercise, SE (B), gum acacia, GA (C), GA + SE (D), adenine (E), adenine + SE (F), adenine + GA (G) and adenine + GA + SE (H), and stained with hematoxylin & eosin (H&E) stain. Sections **A, B, C**, and **D** showed normal kidney architecture and histology. Sections **E** and **F** showed acute tubular necrosis (arrow head) with tubular distention with necrotic material (thin arrows), and many apoptotic cells (thin arrows). Sections G and F showed similar improvement in histological appearance with focal areas of acute tubular necrosis (arrow head), and less dilated tubules (thin arrow).

**Figure 7:**
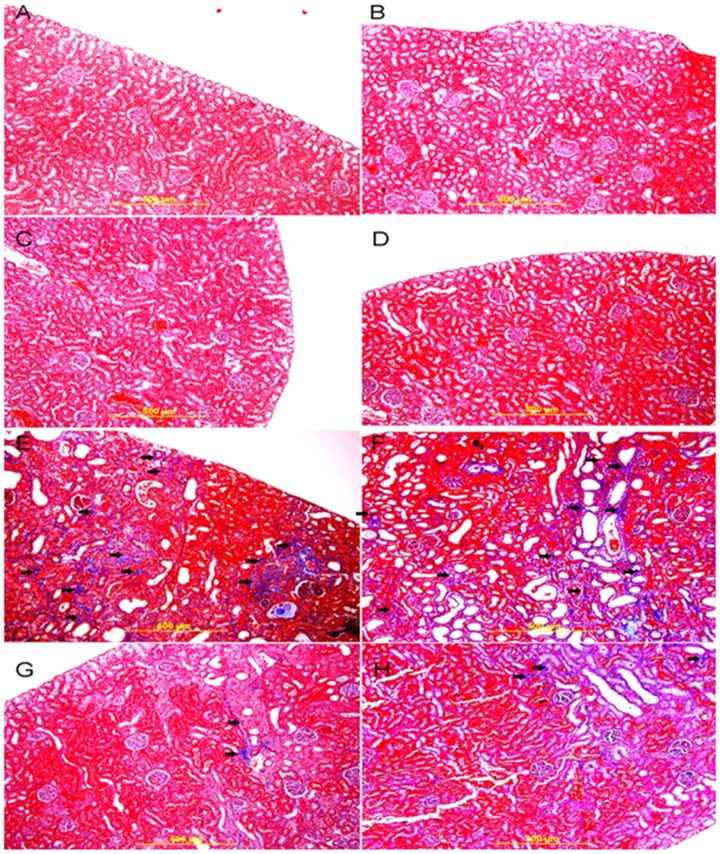
Representative photograph of sections of renal tissue of rats treated with saline (A), saline + swimming exercise, SE (B), gum acacia, GA (C), GA + SE (D), adenine (E), adenine + SE (F), adenine + GA (G) and adenine + GA + SE (H), and stained with Masson trichrome stain. Sections A, B, C, and D showed normal kidney architecture and histology and no evidence of fibrosis. Sections E and F showed large areas of interstitial fibrosis (thick arrows). Sections G and F showed similar improvements in histological appearance with dramatic decrease in fibrosis (thick arrows).

**Figure 8:**
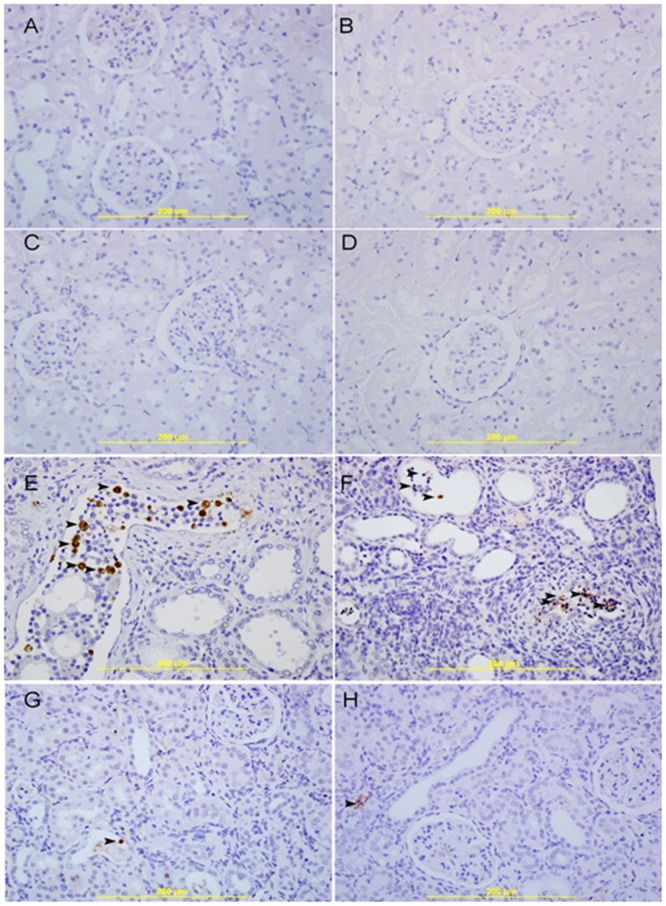
Representative photograph of sections of renal tissue of rats treated with saline (A), saline + swimming exercise, SE (B), gum acacia, GA (C), GA + SE (D), adenine (E), adenine + SE (F), adenine + GA (G) and adenine + GA + SE (H), and analyzed immunohistochemically (anticaspase-3, streptavidin–biotin immunohistochemical method). Sections (**A**), (**B**), (**C**) and (**D**) showed normal kidney architecture and no apoptotic cells. Sections (**E**) and (**F**) both showed acute tubular necrosis with tubular distention and necrotic material and many apoptotic cells showing brown cytoplasmic staining (arrow head). Sections (**G**) and (**H**) both showed a similar degree of improvement in the histological appearance with few focal areas of acute tubular necrosis and very few apoptotic cells showing brown cytoplasmic staining (arrow head).

The kidneys of both sedentary and exercised control and GA –treated rats had normal kidney architecture and histology and were given a score of 0 for necrosis, using H &E staining (Fig 6 A - D). Using the Masson trichrome stain, there was no evidence of fibrosis in these groups (Fig 7, A –D). There was also no evidence of apoptotic cells in the examined sections (Fig 8, A – D).

Fig 6 I and J, Masson trichrome staining showed that in the adenine – treated rats there was a diffuse tubular necrosis in 71.1±7.3% of the examined tissue areas (score 3), tubular distention with necrotic material, loss of brush border of proximal tubules, dilatation of large number of tubules, mixed inflammatory cells infiltration of the interstitium and focal tubular atrophy.

Fig. 1 I &J, Masson trichrome staining showed foci of interstitial fibrosis involving 41.3±5.6% of the examined surface area. The adenine + swimming group showed diffuse acute tubular necrosis in 69.8±9.1% of the examined tissue areas (score 3) showing tubular distention with necrotic material, loss of brush border of proximal tubules, dilatation of a large number of tubules, mixed inflammatory cells infiltration of the interstitium, and focal tubular atrophy (Fig. 6 K and L).

Masson trichrome staining showed foci of interstitial fibrosis involving 42.1±4.9% of the examined surface area (score 2) (Fig. 2F). Many apoptotic cells were seen in Fig.3F. There was no significant histological improvement in necrosis and fibrosis following SE.

The group treated with adenine +GA showed dramatic improvement in the histological appearance when compared with the group treated with adenine alone. There were focal areas of acute tubular necrosis involving 18.2±2.4% of the examined areas (score 1), few tubules showing dilatation, less interstitial inflammatory cells infiltration, less tubular atrophy (Fig.1 M&N), minimal fibrosis of 3.35±0.24%, a score of 1 (Fig.2 G), and few apoptotic cells (Fig. 3G).

The adenine-GA-swimming - treated group showed dramatic improvement in the histological appearance when compared with the adenine –treated group, and showed no significant histological difference from the adenine-GA- treated group (Table 3). There were focal areas of acute tubular necrosis involving 17.8±4.9% of the examined areas (score 1), few tubules showing dilatation, less interstitial inflammatory cells infiltration, less tubular atrophy (Fig.1 O&P), minimal fibrosis of 3.42±0.16% (score 1) (Fig.2 H), and few apoptotic cells(Fig. 3H).

**Table 3:** Evaluation of necrosis and fibrosis in kidneys from rats with chronic kidney disease induced by adenine (A) feeding (0.25% w/w, 5 weeks), and the influence of gum acacia (GA) [15% w/v in drinking water, 5 weeks] with or without swimming exercise (SE) thereon.

Group	% Necrosis	Necrosis Score	% Fibrosis	Fibrosis Score
Saline -treated	0±0^a^	0	0±0^a^	0
Saline + SE	0±0^a^	0	0±0^a^	0
A -treated	71.1±2.9^b^	3	41.3±2.3^b^	2
A –treated + SE	69.8±3.7^b^	3	42.1±2.0^b^	2
GA	0±0^a^	0	0±0^a^	0
GA + SE	0±0^a^	0	0±0^a^	0
A + GA	18.2±0.97^c^	1	3.35±0.09^c^	1
A + GA + SE	17.8±2.0^c^	1	3.42±0.07^c^	1

Values in the table are means ± SEM (n  =  6 rats).

Values with different superscripts are statistically different (*P* less than 0.05).

## Discussion

CKD is known to be a long –term condition that is associated in most cases with physical and psychological symptoms. The former include fatigue, muscle weakness and reduced stamina. It is conceivable that various forms of appropriate exercise can improve these signs and symptoms. It has previously been shown that GA can ameliorate CKD experimentally in rats and mice [16] and clinically in humans [17]. It was of interest, therefore, to find out if there is any interaction between these two variables.

Our results indicated that the body weight of exercised rats did not increase compared with that of sedentary control rats when both were given free access to food. Such exercise is considered of moderate intensity [28]. In the present work we have confirmed an earlier observation that treatment with GA decreases body weight [16], [20] in rats, and also in humans [29]. SE in control rats did not significantly affect the body weight, but it enhanced further the drop in body weights of adenine – treated and GA – treated healthy rats. The body growth depressive action of SE in our rats may be due to a lower intake of feed, although it has previously been reported that energy intake in the hemodialysis patients of Koufaki et al [30] was slightly (5%) but significantly increased.

In this work, the adenine – treated rats exhibited the urinary and plasma profile of several traditional and novel markers of renal damage, as reported by us and others [20], [36], [37]. Most of these were improved in rats given either GA or SE, and even more so in rats given GA and subjected to SE at the same time, supporting our hypothesis that the ameliorative action of GA on adenine- induced CRF is further enhanced by SE. The use of novel urinary and plasma biomarkers has been recently highlighted as being able to detect subtle and early renal changes in both chronic and acute renal injury [38]. In this work, both traditional and novel biomarkers measured in urine and plasma were nearly all in full agreement. Examples of these novel biomarkers used included 8-isoprostane, which is a prostaglandin (PG)-F2-like compound that belongs to the F2 isoprostane class. It is produced *in vivo* by the free radical-catalyzed peroxidation of arachidonic acid, and its concentration is increased in conditions and diseases involving oxidative stress [39]. Urinary 8-oxo-2'-deoxyguanosine (8-oxo- dG) concentration is another measure of oxidative DNA stress [28].

Different modes of exercise, including SE, are established to be beneficial in CKD and its cardiovascular and other complications in humans [27], [31] and animals [31], [28]. The mechanism by which SE ameliorates CKD is not known with certainty, but it has been hypothesized that the basis of the obtained benefits are probably multifactorial [40], and include the beneficial effect of SE on the oxidative status of the tissues. Although there is no unanimity in the literature regarding the influence of exercise on inflammation and oxidative stress, moderate SE is believed to be effective in preventing inflammation and oxidative damage in tissues of rats [32], [33], but severe/acute exercise has been shown to produce the opposite effect in humans and rats [34], [35]. In our present experiments employing moderate SE, we found that SE did not significantly alter the renal concentration/activity of the measured incidence of oxidative stress (except SOD activity, which was increased), probably reflecting the adequacy of the defensive antioxidant oxidative abilities in these animals. Adenine – induced CKD, as reported before, significantly and markedly decreased the anti-oxidants measured [20], [37], an action that was significantly abrogated by either GA or SE given alone, and even more when combined.

In conclusion, we aimed to ascertain experimentally if combining two strategies for mitigating the effects of CKD (viz administration of GA, a nephroprotectant [26] and SE [14] would influence the effects of CKD. Judging by the results of several biochemical and physiological (but not all) parameters measured, there seems to be a significant positive impact in the condition with SE. Therefore, on the whole, these results suggest that the ameliorative action of GA can be enhanced by SE. Previously, it has been reported that significant clinical benefits are obtained from GA treatment in CKD patients who are on a low-protein diet [26]. In future experiments, it would be of interest to see the effect of other modes of exercise with different intensities on the same parameters, and also the possible effect of SE on CKD patients on GA both with and without a low-protein diet.
